# Why reinvent the wheel? Lessons in schistosomiasis control from the past

**DOI:** 10.1371/journal.pntd.0005812

**Published:** 2017-10-26

**Authors:** Clive Shiff

**Affiliations:** Department of Molecular Microbiology and Immunology, Johns Hopkins Bloomberg School of Public Health, Baltimore, Maryland, United States of America; Swiss Tropical and Public Health Institute, SWITZERLAND

## Abstract

Schistosomiasis has been of concern to local health authorities for most of the last century, and in spite of a lack of effective chemotherapy, the disease was dealt with quite effectively in many endemic countries by snail control and environmental management [1]. Much of this work was reported in journals prior to the electronic era but, sadly, seems to have been subsequently ignored. For many years, there followed a global hiatus on schistosomiasis control, and much of the local expertise was lost, but many things have changed more recently, mainly with the advent of generic and affordable praziquantel. With the increased availability of this drug, there has been an increasing interest in readdressing schistosomes as well as other neglected tropical diseases (NTDs). The strategic approach for this had been based almost exclusively on chemotherapy. Recently, however, questions arose about this strategy with evidence that chemotherapy alone was not succeeding [2]. Additional strategies were needed, and the “Towards Elimination of Schistosomiasis” (TES) 2017 Conference in Cameroon stressed an integrated PHASE strategy. This was in line with the WHO-NTD and WHO-AFRO 2014–2020 Regional Strategy on NTDs and directed emphasis on transmission control. Subsequently, this emphasis was discussed in a comprehensive review [3] that stressed the importance of such additions to any elimination programme. In reality, this means focusing on the aquatic snail hosts where and when transmission occurs, defining other risk factors such as water contact and latrine design and identifying improved sanitation and health education as essential components for elimination. For schistosomiasis reduction during the mid-20th century, transmission control was used extensively, but these facts are not well reported. Recent reviews have attempted to cover previous research [4,5], but sadly, they have left major knowledge gaps, particularly from Africa. These omissions also occurred in a recent WHO pamphlet on molluscicides [6]. Sadly, search engines used to retrieve information appear to miss much done by 5 African research institutes active from 1950 to 1990. It seems appropriate to take a look back to a time when fieldwork was a focus of research and transmission control was emphasised.

## The molluscan role

Aquatic snail vectors were the target of intervention and research efforts in Egypt and in East, Central, and South Africa as well as Brazil and the Caribbean; they focused on these snails, and there is much to learn from these interventions that could invigorate current programmes. The objective here is to consider aspects of research, primarily in Africa, that have been missed by reviewers but nevertheless could help modern interventions succeed. This discussion is based on some 20 years of work in what is now Zimbabwe, interacting personally with the other colleagues working with planorbid snails and their relationships to *Schistosoma haematobium* and *S*. *mansoni*.

The study of aquatic snails requires an appreciation of issues related to limnology [7]. In the waterbody, snails are motile and can select locations that are suitable for them. Pulmonate gastropods can crawl below the meniscus, where the temperature may be warmer than 5 cm down. In winter, the surface meniscus is their preferred habitat, but if disturbed, the snails can plummet to the bottom of a pond quickly. Snails will adjust their specific gravity at will and can select an area where the preferred temperature exists [8]. In different seasons, schistosome miracidia orientate to these areas where the hosts snails are likely to be found [9]. Fast-flowing water disturbs this stratification and, if in excess of 0.5–1.0 m/sec, will dislodge some individuals. It is important to understand these properties; they represent many of the transmission foci, and focal control of snails can impact transmission [10].

Different snail species have different requirements, but currently there is a profound ignorance of the taxonomy of intermediate host snails. Little emphasis is placed on the snail populations in research design, and there are few malacologists available to advise. There is little malacology discussed in the latest WHO publication on molluscicides [6]. Not all aquatic snails transmit schistosomes, and some associations are very specific [11]. The current literature reports *Bulinus globosus* transmitting *S*. *haematobium* in sub-Sahelian Africa. This is not strictly correct; for clarity, the name should be *B*. *(Physopsis)* spp. with several species in this subgenus, including *B*. *africanus*, *B*. *globosus*, *and B*. *nasutus* [11], which differ morphologically and also by molecular analysis [12]. Not all are vectors, and some are very specific in this relationship. Species in the subgenus will host *S*. *haematobium* and the bovine schistosome *S*. *mattheei* from Southern and East Africa [13] as well as the southern parts of West Africa but will not host *S*. *haematobium* or *S*. *bovis* from Egypt, the Middle East, and countries in the northern part of the Sahel region. The northern strain of *S*. *haematobium* infects *B*. *(Bulinus) truncatus*. This is all stated in Brown’s monograph [11], and it is not trivial systematics. Members of the *B*. *(Bulinus)* spp. group, e.g., *B*. *tropicus*, are distributed all over Africa, but this latter species will not serve as intermediate host to *S*. *haematobium*, whether in northern or southern Africa. Sadly, few people now working on schistosomes in Africa can distinguish between these species or between the species groups discussed above. In river systems, *B*. *tropicus* and *B*. *globosus* are frequently sympatric [11]. They can be distinguished through the shell morphology, but the differences can be subtle, and it is important to differentiate between these. Obviously, snail taxonomy should be explained to workers involved in schistosomiasis control. These problems do not arise with *S*. *mansoni* because *Biomphalaria* spp. are more easily identified, and all forms are potential vectors for the parasite.

## Molluscicides

Niclosamide was used extensively in Zimbabwe in programmes to control bilharzia and liver fluke [14]. Elsewhere in Africa, molluscicides were tested in a 2-year study in Tanzania by Webbe [15] and in a composite study in St Lucia reported in Peter Jordan’s book [16]. Irrigation schemes were of considerable concern and were the main subject of applied research. A comprehensive annual mollusciciding operation in a 20,000 ha series of irrigation estates in Zimbabwe reduced transmission effectively over the entire irrigation area. The work was funded by farmers and repeated annually with continued success [17]. These interventions were cost effective in productivity and improved the health of farm residents.

However, when applying toxic substances in natural water systems, the environmental impact is important. No current review on molluscicides has dealt with this, but it was and is a matter of concern. A comprehensive study on molluscicide toxicity in Rhodesia (now Zimbabwe) reported by Harrison and Rattray [18] observed insignificant change in the numbers and species of plankton as well as benthic and pelagic species, fish, and amphibians following systematic application of niclosamide. Populations were sampled prior to and 1 week after the application of molluscicide where routine blanket spraying of niclosamide was underway. Similarly, after 10 years of annual mollusciciding over 20,000 ha irrigation areas of the Zimbabwe lowveld, there was no apparent negative impact on aquatic fauna and flora over the entire area (personal communication, A. C. Evans to C. Shiff).

Does mollusciciding have to be done continuously, especially when seasonal fluctuations of parasite infectivity are clear? No! But it does require detailed knowledge of the transmission foci to be effectively implemented. For example, it was shown in South Africa that infected snails kept out doors shed cercariae with strong seasonal affects [19]. This was repeated at the Blair Laboratory in Zimbabwe in a 12-month study in both highveld (above 1,250 m) and lowveld (below 800 m) areas. We demonstrated that *B*. *globosus* snails infected with *S*. *haematobium* and *Biomphalaria pfeifferi* infected with *S*. *mansoni* went into a “diapause” if exposed to miracidia from late summer through winter [20]. This adaptation to changing temperature caused an increase in the parasite incubation period as winter approached and a cessation of cercariae shedding from May until August, when waterbodies warmed in spring. The diapause ended with a massive release of infective cercariae. Human exposure at this time would produce heavy infection and suggested that winter application of molluscicide to focal transmission points might preclude this and reduce transmission [21]. This hypothesis was tested in a 5-year study in a rural area east of Harare. Communities were aware of bilharzia and were prepared to collaborate [21]. Working with school authorities, local school children ages 6 to 9 and 10 to 14 were examined thrice over 5 years, initially prior to the application of molluscicide, then after 3.5 years, and again 1 year later, thus covering a 5-year period. All known water contact points used by people (drawing water, ablution, laundry, and recreation) were surveyed and sprayed with molluscicide once each winter from 1974 to 1977. Mass drug administration was not possible because no appropriate drug for apparent asymptomatic infection was available. Initially, the prevalence in all 6 schools was similar across the study area. Three schools were situated in the treatment area, and 3 were in another similar but more distant area. The prevalence of *S*. *haematobium* declined appreciably in each age group in the 3 schools serving children where annual contact-point spraying was carried out, while it remained similar or slightly increased in the 3 schools in the adjacent “control” districts [21]. This suggests that transmission was interrupted by annual focused spraying. This work was done without the availability of a global positioning system (GPS) or satellite imaging. It certainly could be feasible today [22].

## Sanitation and hygiene

**Fig 1:**
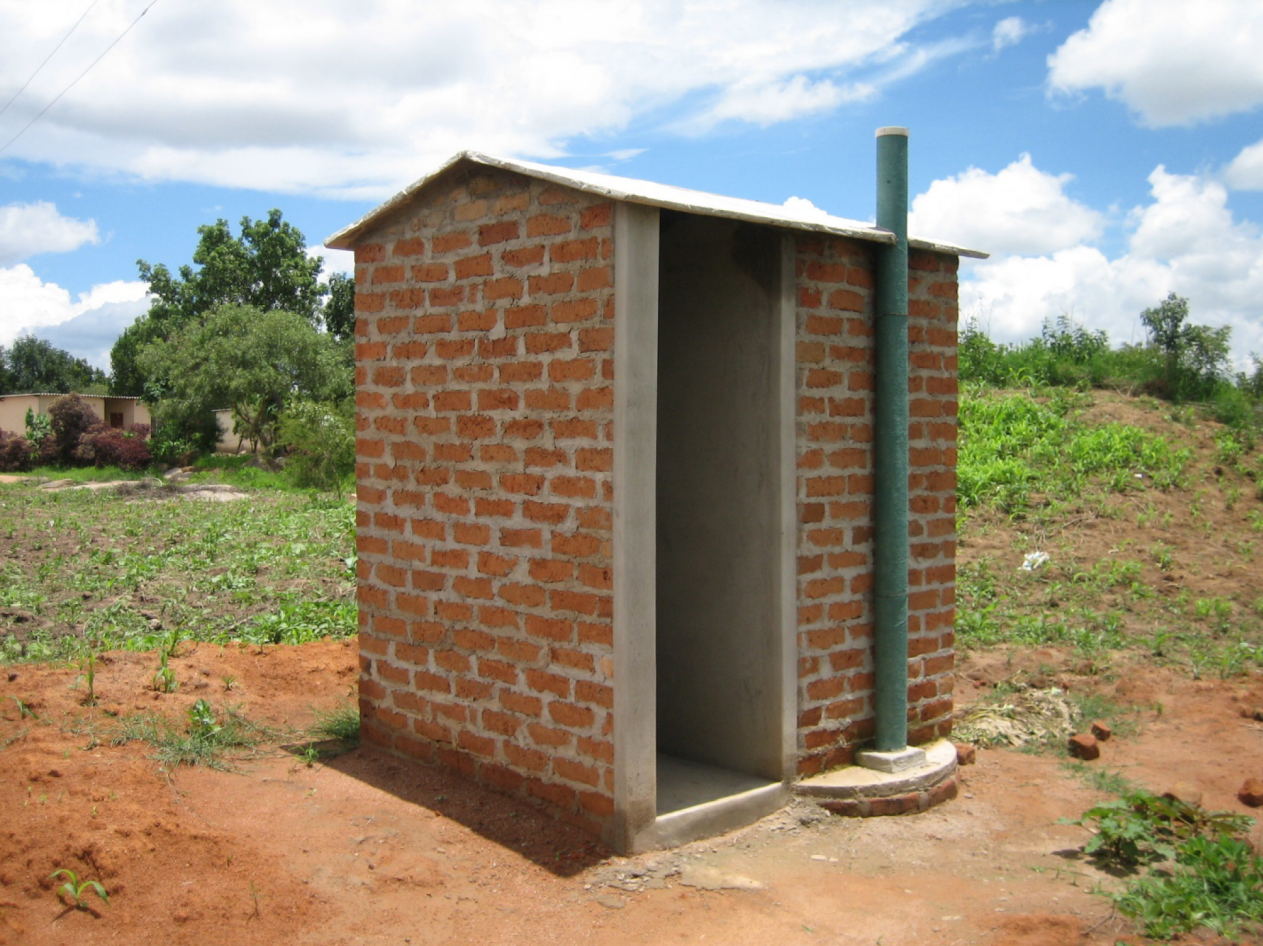
The Blair VIP latrine. Simple construction of ideal family latrine used extensively in Zimbabwe. Often used also as an ablution facility as well as a latrine; this is adapted for individual family use. The flue (vent) exhausts the odour and also diverts flies. The pit is approximately 5 m deep. Photograph: Dr. Peter Morgan.

In the long run, a reduction of the transmission of schistosomiasis and other soil-transmitted helminths will require effective sanitation and improved water supplies to reduce the need to use and pollute natural river water for domestic purposes. Field studies observing human water contact clearly indicate routinely utilised pools and stream banks [23]. Almost all domestic contact is by women and children and occasionally fishermen and bathers, thus providing opportunities to infect snails and to acquire infections from cercariae [23]. While faecal contamination of the area can be reduced by the provision of adequate latrines, it is difficult to prevent children and others from urinating in the water. However, in order to promote hygiene in schools and among the community, proper facilities are necessary. Clean, odourless latrines are appropriate [24]. The Blair VIP latrine was designed and developed in Zimbabwe [25]. Properly constructed with a concrete slab painted with bitumen and provided with a vent flue that makes the unit odourless, the latrine is frequently used for bathing as well as for sanitary purposes. The addition of water from the bath going into the pit keeps the pit moist and causes anaerobic decomposition of faeces (see examples at www.aquamor.info). A latrine could serve a family of about 6 for about 20 years before it filled up. An excellent manual by author Peter Morgan [25] can be obtained online (at http://www.susana.org/_resources/documents/default/2-1177-buildersmanualforubviplonguniversalventpipe.pdf). Such latrines need to be promoted on a basis of at least 1 per family. This ensures that the facility will be kept clean and used as needed. However, this design is seldom seen now in rural areas because these facilities are probably not promoted adequately by health personnel. Perhaps they could be better informed. An example of such a latrine is shown in Fig 1.

## Conclusion

In light of the interest in transmission control for schistosomiasis [2,4,26,27], it makes sense to look back in the literature to a time when the only intervention available, certainly with schistosomes, was transmission control. There is much to be learned from past work, which is scattered through the literature and is difficult to access by modern electronic retrieval systems, but it is worth the effort. It is clear there was success in transmission control, and past experience will help in planning modern interventions.
